# Effect of Different Modes of Delivery on Cord Blood Oxidative Stress Markers

**Published:** 2013-12

**Authors:** Daniel Adebode ADEKANLE, Dolapo Pius OPARINDE, Adeniran Samuel ATIBA, Akinyemi Akinsoji AKINTAYO

**Affiliations:** 1 Department of Obstetrics and Gynaecology, Ladoke Akintola University of Technology Osogbo, Osun State, Nigeria;; 2 Department of Chemical Pathology, Ladoke Akintola University of Technology Teaching Hospital, Osogbo, Osun State, Nigeria;; 3 Department of Chemical Pathology, Ekiti State University, Ado-Ekiti, Ekiti State, Nigeria;; 4 Department of Obstetrics and Gynaecology , Ekiti State University, Ado-Ekiti, Ekiti State, Nigeria

**Keywords:** oxidative stress, cord blood, modes of delivery, free radical, newborn

## Abstract

**Background:**

Normal pregnancy has been associated with oxidative stress injury. Oxidative stress has been linked with poor perinatal outcome and birth asphyxia. The severity of this oxidative stress in newborn may be related to stress of different modes of delivery.

**Methods:**

Eighty seven newborn babies were recruited in both labour ward and operating theatre of Ladoke Akintola University of Technology Teaching Hospital, Osogbo, Nigeria. Fourty one of them was delivered via spontaneous vertex delivery, 26 via emergency caesarean section and the rest, 20 were delivered through elective caesarean section. Cord blood sample was collected from new born babies immediately after delivery. Plasma was extracted and used for the laboratory measurement of total antioxidant status, malondialdehyde and Uric acid.

**Results:**

There were no significant (*P*>0.05) changes among the studied groups in mean plasma levels of malondialdehyde, total antioxidant status and uric acid. However, a trend was observed in these parameters. Mean plasma total antioxidant status/mmol/l was observed to be highest in subjects delivered through ECS (2.35 ± 0.05) and lowest in subjects delivered through SVD (2.03 ± 0.08). Similarly mean plasma UA/mg/dl was also observed to be highest in subjects delivered through ECS (3.61 ± 0.16) lowest in those delivered through SVD (3.49 ± 0.71). The highest mean plasma level of MDA/µmol/l was found in subjects delivered through SVD (5.78 ± 1.56) while the lowest was found in subjects delivered through ECS (5.01 ± 1.21).

**Conclusion:**

There is no significant relationship between oxidative stress markers in neonate and the mode of delivery.

## INTRODUCTION

Oxidative stress is known to occur in apparently normal pregnancy, even in the absence of complications (1). Exposure to significant oxidative stress in the intrauterine life has been linked to intrauterine growth retardation (2, 3), intrauterine fetal death (4) as well as poor perinatal outcome (5). Fetuses exposed to oxidative stresses in the intrauterine life are at increased risk of birth asphyxia and this may be worsened by the stress of labour and delivery. Babies are delivered either vaginally or through caesarean delivery depending on the circumstances surrounding the pregnancy from conception through labour (6). Each of these modes of delivery has its own effects both on the baby and the mother.

Bearing in mind the stress of labour as the fetus navigates the birth canal, an assumption can be made that free radical may be generated more in women and babies delivered through spontaneous vertex delivery (SVD) than those delivered by caesarean section (CS), especially planned CS. The event that may lead to emergency caesarean section (EMCS) may however be associated with generation of more free radical and increased consumption of antioxidants. Babies delivered through elective caesarean section (ECS) if the indication does not relate to oxidative stress injury may be most free of this injury.

Based on the above facts and hypothesis this study was therefore designed to measure total antioxidant status (TAS), uric acid (UA) and malondialdehyde (MDA) in cord blood of babies delivered through this different modes of delivery. Plasma MDA has been shown to be an index of free radical (oxidant) injury on membrane lipids. Status of these antioxidants and oxidant will give an idea of cord blood oxidative stress of these babies. Uric acid may be a marker of oxidative stress (7), and may have a potential therapeutic role as an antioxidant (8) .On the other hand, like other strong reducing substances such as ascorbate, uric acid can also act as a pro-oxidant (9, 10). Thus, it is unclear whether elevated levels of uric acid in diseases associated with oxidative stress are a protective response or a primary cause.

## MATERIALS AND METHODS

This is a prospective study that was carried out in Ladoke Akintola University of Technology Teaching Hospital, Osogbo, Nigeria. Subjects were recruited randomly in both labour ward and operating theatre of this institution. Eighty seven new born babies were recruited into the study. Fourty one of them was delivered via spontaneous vertex delivery (SVD), 26 were delivered via emergency caesarean section (EMCS) and the rest, 20 were delivered through elective caesarean section (ECS). Mothers of these babies were not known hypertensive; diabetic, active smokers and none had preeclampsia. Babies of women that had their labour prolonged for more than 16 hrs were also excluded. None of the babies was among a set of multiple gestations and those with obvious congenital malformation were excluded. Informed consent was taken from the mothers before cord blood was taken. Ethical clearance was gotten from ethical and research committee of Ladoke Akintola University of Technology Teaching hospital, Osogbo, Nigeria, our local research committee.

Cord blood sample was collected from new born babies immediately after delivery. Immediately after delivery, the umbilical cord was clamped at two points. Sterile needle was inserted into a superficial umbilical vessel in between the two clamps and 5 ml of blood was drawn. This was dispensed into lithium heparin containing specimen bottles. The sample was however, centrifuged at 3000 g for 10 minutes and supernatant (plasma) was extracted into plain specimen bottle. The plasma was therefore kept frozen until laboratory analysis. This plasma was used for the laboratory measurement of TAS, MDA and Uric acid. Total antioxidant status was measured using method by Koracevic *et al* (11). A standardised solution of Fe-EDTA complex reacts with hydrogen peroxide to give hydroxyl radical, a reactive oxygen species. These reactive oxygen species degrade benzoate to produce thiobarbiturate reacting substances (TBARS). The antioxidant from added sample causes suppression of the production of TBARS. Therefore the decrease in the concentration of TBARS as a result of antioxidants is measure spectrophotometrically and serves as concentration of antioxidants present in the biological sample added. Plasma MDA was measured using method by Satoh *et al* (12). The principle is based on malondialdehyde in the plasma reacting with thiobarbituric acid (TBA) in acidic medium to form a pink colour complex measured spectrophotometrically. The deeper the colour generated the higher the concentration of malondialdehyde. Plasma uric acid was measured using ready to use commercially manufactured kit by Fortress Diagnostics Limited, Unit 2C Antrim Technology Park, Antrim NT41 IQS, United Kingdom. The principle is that uric acid in plasma is converted to allantoin and hydrogen peroxide in the presence of uricase. Hydrogen peroxide, in the presence of peroxidase enzyme is converted to Quinone-dimine dye. This colour complex is measured spectrophometrically.

Continuous and categorical variables were displayed as means ± SD and percentages respectively. Differences between continuous variables were analyzed using Student’s t test. Differences among ≥ 2 groups were compared using the Analysis of Variance (ANOVA) test. All data analyses were done using the Statistical Programme for Social Sciences (SPSS) version 16.0 (SPSS Chicago Inc., IL, and U.S.A.). *P*-value<0.05 was regarded as significant.

## RESULTS

A total of 87 subjects were recruited. Seventy two percent of the recruited subjects were primigravida while 28% were multigravida. The mean ages of the recruited mothers were 33.28 years, 28.50 years and 34.00 years for SVD, EMCS AND ECS groups respectively. Other considered babies and mothers characteristics are as presented in Table 1. Indications for CS for those delivered either through ECM or EMCS are as presented in Table 2 below.

**Table 1:** Babies and Mothers Characteristics

	SVD	EMCS	ECS

Mean Mothers’ Age (year)	33.28 ± 5.5	28.50 ± 3.23	34.00 ± 3.45
Mean Mothers’ Weight (kg)	82.00 ± 10.34	79.00 ± 9.34	81.00 ± 10.23
Mean Weight of Babies (kg)	2.90 ± 0.23	3.00 ± 0.32	2.85 ± 0.34
Mean Weight of Placenta (kg)	0.45 ± 0.03	0.49 ± 0.02	0.48 ± 0.05
Mean gestational Age at delivery(weeks)	37.52 ± 2.13	37.50 ± 3.11	39.14 ± 1.23
Duration of labour (hr)	8.00	10.0 (before CS)	-
Median Apgar Score (1^st^ minute)	8	7	8
Median Apgar Score (5^th^ minute)	10	10	10
Mean maternal SBP	132 ± 5.03	128 ± 6.03	120 ± 10.23
Mean maternal DBP	72 ± 3.03	68 ± 7.12	61 ± 3.03

**Table 2:** Indications for Caesarean section among the study groups

Indication	EMCS (n=26)		ECS (n=20)	

Fetal distress	3	11.54%	-	-
Prolong labour	-	-	-	-
Antepartum Haemorrhage	4	15.38%	-	-
Malpresentation	16	61.54%	10	50.00%
Prolong pregnancy	-	-	5	25.00%
Abnormal lie	3	11.54%	5	25.00%

Table 3 shows comparison of biochemical parameters among the study groups. Mean plasma total antioxidant status was observed to be higher in subjects delivered through EMCS (2.12 ± 0.10 mmol/l) than those delivered through SVD (2.03 ± 0.08 mmol/l). Mean plasma TAS was also observed to be higher in subjects delivered through ECS (2.35 ± 0.05 mmol/l) than those delivered through SVD (2.03 ± 0.08 mmol/l). When TAS also was compared between subjects delivered through EMCS and ECS, mean plasma level was found to be higher in subject delivered through ECS (2.35 ± 0.05 mmol/l) than those delivered through EMCS (2.12 ± 0.10 mmol/l) However, these differences in mean plasma TAS levels were not statistically significant; *p*>0.05. Mean plasma was observed was observed to be higher in subjects delivered through EMCS (3.95 ± 0.22 mg/dl) than those delivered through SVD (3.49 ± 0.71 mg/dl). Also mean plasma value of UA was observed to be higher in subjects delivered through ECS (3.61 ± 0.16 mg/dl) than those delivered through SVD (3.49 ± 0.71 mg/dl). Similarly mean plasma value of UA was observed to be higher in subjects delivered through ECS (3.61 ± 0.16 mg/dl) than those delivered through EMCS (3.55 ± 0.22 mg/dl). Mean plasma level of MDA was found to be higher in subjects delivered via SVD (5.78 ± 1.56 µmol/l) than those delivered through EMCS (5.34 ± 1.86 µmol/l). Also mean plasma level of MDA was found to be higher in subjects delivered through SVD (5.78 ± 1.56µmol/l) than those delivered through ECS (5.01 ± 1.21µmol/l). In the same vein, mean plasma MDA was found to be higher in subjects delivered through EMCS (5.34 ± 1.86 µmol/l) than those delivered through ECS (5.01 ± 1.21µmol/l). However, these differences in mean plasma MDA levels were not statistically significant; *p*>0.05.

**Table 3:** Comparison Of Mean ± SD Among the Study Groups

Variables		Mean ± SD	*P*-value

TAS/mmol/l	SVD V EMCS	2.03 ± 0.08 V 2.12 ± 0.10	*p*>0.05
	SVD V ECS	2.03 ± 0.08 V 2.35 ± 0.05	*p*>0.05
	EMCS V ECS	2.12 ± 0.10 V 2.35 ± 0.05	*p*>0.05
Uric Acid/mg/dl	SVD V EMCS	3.49 ± 0.71 V 3.55 ± 0.22	*p*>0.05
	SVD V ECS	3.49 ± 0.71 V 3.61 ± 0.16	*p*>0.05
	EMCS V ECS	3.95 ± 0.22V 3.61 ± 0.16	*p*>0.05
MDA/μmol/l	SVD V EMCS	5.78 ± 1.56 V 5.34 ± 1.86	*p*>0.05
	SVD V ECS	5.78 ± 1.56 V 5.01 ± 1.21	*p*>0.05
	EMCS V ECS	5.34 ± 1.86 V 5.01 ± 1.21	*p*>0.05

SVD, spontaneous vertex delivery; EMCS, emergency caesarean section; ECS, elective caesarean section.

**Figure 1:**
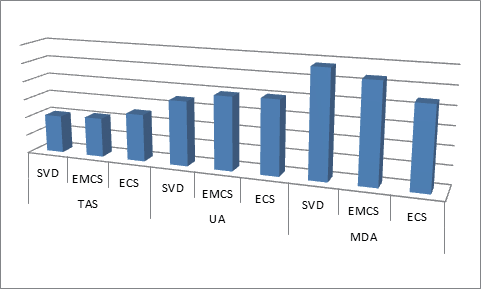
Trend in Differences of Biochemical Parameters Among the Study Groups. SVD, spontaneous vertex delivery; EMCS, emergency caesarean section; ECS, elective caesarean section.

The trend in these biochemical parameters are that, the highest mean TAS level was observed in subjects delivered through ECS while the lowest was found in the subjects delivered through SVD. Contrarily, the highest plasma mean level of MDA was observed in subjects delivered through SVD and the lowest level was observed in subjects delivered ECS. This has presented in Fig. 1.

## DISCUSSION

Oxidative stress in biology and medicine occurs when generation of free radicals (oxidants) overwhelm the available antioxidants (13). Also it could be as a result of insufficient antioxidants in the system. This occurs even when there is minimal generation of free radicals. Majority of these free radicals like in the group reactive oxygen species have a damaging effect on cellular organelles like polyunsaturated membrane lipids (14). Free radical attack on membrane lipid produces malondialdehyde (MDA) as one of the intermediate products of these dangerous reactions (14). Therefore, to measure oxidative capacity of these free radicals, malondialdehyde measurement from any biologic sample is used (15). This study observed no significant differences in the plasma levels of MDA in cord blood of babies among the studied groups. This means that free radical generation in babies is of the same significance despite passing through various stressful conditions through their different modes of delivery. However, trend of changes in plasma MDA levels among the study groups though not statistically significant may predict free generation to be highest in babies delivered through SVD and lowest in babies delivered through EMCS. This means that babies delivered through SVD had most free radical attack on membrane lipids than other study groups. However, this may not be true of babies whom their mothers had prolong labour before delivery. Although none of our subjects experienced prolong labour. Babies delivered through EMCS also may have more free radical generation than those delivered through SVD if the events that lead to their emergency CS faviour free radical generation. Generally, cord blood malondialdehyde has been found to be an indicator of perinatal oxidative stress (6). A study by Chitra *et al* in 2005 (16) and Argüelles *et al* in 2006 (17) have shown also that free radical generation in mother is associated with what observed in the newborn. This observation may not mislead in the interpretation of the result of our study because clinical conditions like pre-eclampsia that have been linked to free radical generation were well excluded. All recruited pregnant women in this study had apparently normal pregnancy. They were equally exposed to observed free radical generation of normal pregnancy.

Antioxidants play an important role in preventing the formation of and scavenging of free radicals and other potentially toxic oxidizing species. There are three categories of antioxidant species: antioxidant enzyme systems (glutathione reductase, peroxidase, catalase etc.), small molecules like antioxidant vitamins (ascorbate, uric acid, vitamin E, etc.) and proteins (albumin, transferrin, etc.). Measurement of the combined non-enzymatic antioxidant capacity of cord blood sample provides an indication of the overall capability to counteract free radical (reactive oxygen species; ROS), resist oxidative damage and combat oxidative stress-related diseases. In some cases, the antioxidant contribution of proteins is desired whereas in other cases only the contribution of the small molecule antioxidants is needed. Total antioxidant status (TAS) measurement gives an idea of majority of enzymatic antioxidants in the system. However, one cannot take away additive importance of measuring these antioxidants individually. No significant changes observed in total antioxidant status among our study groups may well explain the above findings on plasma MDA. This means that there was no significant oxidant (free radical) production to stimulate production or consumption of available antioxidants. This is contrary to the findings from previous studies (18, 19). Mutlu *et al* in 2011 (18) observed a lower lipid peroxidation in newborn delivered through CS than those delivered through vagina delivery. Mine *et al* (19) in their study observed oxidative stress to be more in babies delivered through vagina delivery than elective CS. Our finding may be different from the study of Mutlu *et al* and Mine *et al* probably because of compensatory antioxidant that counteracts free radical when produced. However, it will be interested to also mention the trend of changes though not statistically significant observed among the studied groups. It predicts that babies born through ECS have highest levels of total antioxidant capacity and then babies born through SVD. This could explain the consequent consumption of antioxidants in counteracting free radical damage and generation as observed in MDA levels above. Babies delivered through EMCS had lower total antioxidant status than those delivered through ECS. It means antioxidant consumption was more in babies delivered through EMCS. Events of labour mothers were exposed to before a decision to have emergency CS done might have predisposed them to generating more free radicals. This in a way has been proven by the work of Schulpis *et al* in 2006 (20), they observed a lower total antioxidant capacity post vaginal delivery of women that had both normal and prolong labour. Therefore, consumption of antioxidant comes into play to counteract effects of these free radicals.

Uric acid is a major antioxidant in the human plasma (8). It correlates well with conditions associated with oxidative stress (8). It should be emphasized that uric acid may function either as an antioxidant mainly in the plasma (8) or pro-oxidant mainly within the cell (10). Our study observed uric acid status only in the plasma, therefore its antioxidant properties was considered in this study. Like the antioxidant status, no significant changes observed in plasma uric acid among the study groups could be as a result of non significant availability of oxidants to work on. In the same vein, similar trend in changes as in total antioxidant status were also observed. This can also be as a result of consequent compensation from plasma levels of free radical (oxidants). The expected low serum level of uric acid seen in normal pregnancy probably because of increase urate clearance (18) should however be put into consideration (21). This low level in mothers may be expectedly passed on to babies. However, this may not be so because of the increased uric acid production in foetus (18). The lower level of UA found in cord blood of babies delivered through SVD may suggest its usage in counteracting free radical production.

## CONCLUSION

There is no significant difference in oxidative stress markers in subjects born through different modes of delivery.

## LIMITATION

This study did not look into these oxidative stress markers in the subjects’ mothers before delivery. The sample size also was small. It is our hope that, in future study, these markers and more should be done in both cord and maternal specimen with a larger sample size.

## ABBREVIATIONS

CS: Caesarean Section
SVD: Spontaneous Vertex Delivery
EMCS: Emergency CS
ECS: Elective CS
